# Rosai-Dorfman disease in the differential diagnosis of cervical lymphadenopathy

**DOI:** 10.1016/S1808-8694(15)30616-9

**Published:** 2015-10-18

**Authors:** Daniele Cristine Gomes Pinto, Tatiana de Aguiar Vidigal, Bruno de Castro, Bruno Hollanda dos Santos, Nicodemos José Alves de Sousa

**Affiliations:** 1 Otorhinolaryngologist.; 2 Otorhinolaryngologist.; 3 Otorhinolaryngologist, assistant physician of the Otorhinolaryngology and Head & Neck Surgery Unit, Santa Casa de Belo Horizonte, MG.; 4 Otorhinolaryngologist, assistant physician of the Otorhinolaryngology and Head & Neck Surgery Unit, Santa Casa de Belo Horizonte, MG.; 5 Otorhinolaryngologist, master's degree in otorhinolaryngology, UNIFESP. Head of the Otorhinolaryngology and Head & Neck Surgery Unit, Santa Casa de Belo Horizonte, MG. Full professor of Otorhinolaryngology, Faculdade de Ciências Médicas de Minas Gerais. Santa Casa de Misericórdia de Belo Horizonte MG.

**Keywords:** cervical lymphadenopathy, rosai-dorfman disease

## Abstract

Rosai-Dorfman Disease or Sinus Histiocytosis with Massive Lymphadenopathy (SHML) is a rare benign disease of unknown etiology, which presents with cervical lymphadenopathy. It is usually seen in younger patients. The extranodal form affect various regions of the head and neck, and is more common in patients with immune abnormalities. It is a self-limited and seldom life-threatening disease, rendering therapy unnecessary in most cases. For those who require therapy because of persistent or worsening symptoms, treatments modalities include surgery, chemotherapy, radiotherapy and steroids. The authors describe one case of a 43-year-old man with bilateral cervical masses, nasal obstruction, fever, weight loss and decreased vision with 6 months duration. As his social history was positive for tobacco and alcohol use, the initial diagnosis was a possible rhinopharyngeal malignant tumor. Medical investigation established the diagnosis of SHML. After therapy, the 6-month follow-up evidenced the patient's clinical improvement, although cervical masses persisted. The clinical presentation, histological features, pathogenesis and treatment of this case are discussed.

## INTRODUCTION

Sinus histiocytosis with massive lymphadenopathy (SHML) was described in 1969 by Rosai and Dorfman in a report of four cases;^1^ the same authors characterized this disease in greater detail in 1972.^2^ It is a rare, benign lymphoproliferative condition presenting with painless cervical lymphadenopathy, fever, leukocytosis with neutrophilia, increased hemosedimentation rate and polyclonal hypergammaglobulinemia.^3^, ^4^ Other lymph node groups may be involved; 30-40% of cases have extranodal disease.^1^, ^2^, ^3^, ^6^ This condition generally affects children and young adults;5 the mean age at presentation is 19.7 years.^4^ It affects equally white and black people, and is less frequent in Asians. The disease is found worldwide and appears to affect males more than females, in a 1.4:1.0 ratio.^6^ Its etiology remains uncertain. Possible causes include altered immune responses and infections by agents such as varicella-zooster and other herpetic viruses, Epstein-Barr and cytomegalovirus, Brucella and Klebisiela.^4^ Treatment is controversial. Other modalities have been attempted, such as surgery, antibiotics, radiotherapy, chemotherapy and steroids, sometime in combination; none has provided consistent results. We present a case that was diagnosed and treated in our clinical unit, and make a brief review of the literature.

## CASE REPORT

GCO, a male patient aged 43 years, sought the otorhinolaryngology and head & neck surgery unit presenting bilateral neck masses, nasal block, significant weight loss, malaise, evening fever and loss of vision. These symptoms had progressed during the past 6 months. There was no nasal bleeding, odynophagia or dysphonia.

**Figure 2:**
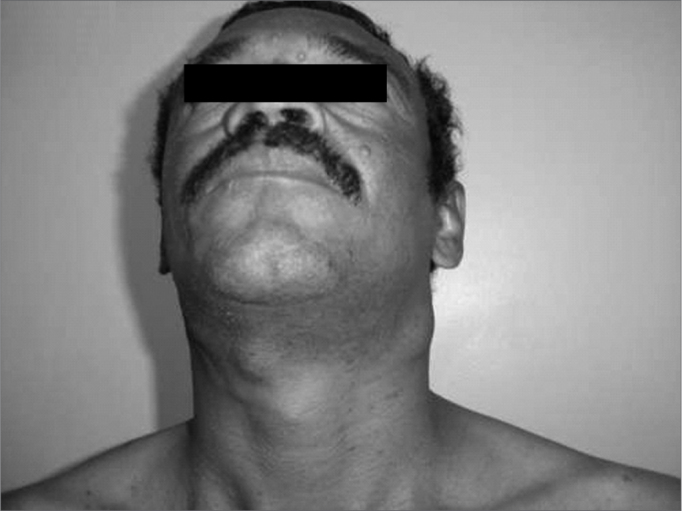
Cervical lymph node enlargement.

The physical examination showed bilateral neck nodules of different sizes, as shown in figure 2. The nodules were coalescent, adhered to deeper planes and painless. There were no other findings.

Fine needle aspiration biopsy (FNAB) was done of a nodule. Other exams were nasal endoscopy, computed tomography (CT) of the neck, a complete blood count, hepatic function tests, screening for autoimmune diseases, VDRL and HIV serology, and testing for toxoplasmosis. These exams revealed leukocytosis and neutrophilia. Anti-HIV, VDRL and serology for toxoplasmosis were negative. The FNAB was non-conclusive; there were lymphocytes, occasional neutrophils and no neoplastic cells.

**Figure 1:**
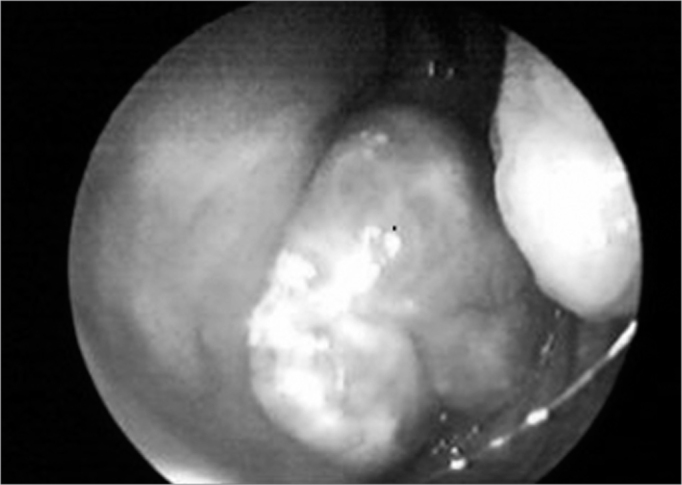
Endoscopic image of a tumor in the cavum.

Nasal endoscopy showed a large lobulated tumor in the cavum (Figure 1), which occluded most of the choanae. CT of the neck showed an expanding solid lesion in the retropharyngeal and parapharyngeal space, projecting into the nasopharyngeal lumen. There was also cervical and submandibular lymph node enlargement.

The tumor was biopsied under endoscopic view. Histology showed a diffuse submucosal infiltration by plasma cells containing lymphocytes within their cytoplasm.

While these exams were done, the patient worsened progressively, presenting bilateral axillary and inguinal lymph node enlargement, diffuse facial skin lesions and further loss of vision.

Histological findings suggested extranodal Rosai-Dorfman disease.

Corticosteroid therapy was initiated (prednisone 1mg/kg/day) and adequate clinical control.

The 6-month follow-up showed that the patient improved significantly, but the neck masses regressed only slightly.

## DISCUSSION

SHML is a rare cause of lymph node enlargement in children and young adults; 80% of cases occur under the age 20 years. Many cases of extranodal involvement have been described since 1969. Apparently, there is no relation between lymph node and extranodal disease; they may even occur separately.^2^

The etiology is uncertain, although agents such as the Epstein-Barr or herpes viruses are important in the pathogenesis.^3^

The onset of SHML is typically insidiously; the active phase is prolonged, there may be spontaneous remission and subsequent relapses.^3^ Factors that determine recurrences and is frequency are not well understood. Deaths have been reported due to infiltration of vital organs, such as the liver.^4^ The neck lymph nodes are the most frequently involved, followed by inguinal, axillary and mediastinal lymph nodes.^5^ The most common extranodal sites are the skin, the upper respiratory tract and bones. Head and neck involvement - about 22% of extranodal disease^4^, ^5^ - include involvement of the nasal cavity, the paranasal sinuses, the nasopharynx, submandibular glands, the parotid, the larynx, the temporal bone, the intratemporal fossa, the pterygoid fossa, the meninges and the orbit.^5^ The skin is commonly affected; half of these patients have another associated extranodal site. Orbit and ocular glove involvement have been reported, usually as a retroorbitary mass and proptosis.^6^

The differential diagnosis of extranodal SHML may be a challenge, and is based on the clinical and histological examination. Histology shows typical features, such as diffuse lymphoplasmatic infiltration, Russel bodies, foamy histiocytes and histiocytes with phagocytosed lymphocytes within the cytoplasm (emperipolesis). Immunohistochemical features include positive S-100, alpha-antichymotrypsin and CD1a and CD68 antigens.^4^, ^5^ Imaging (CT and magnetic resonance imaging) may be used to assess disease extension. If there is cervical lymph node enlargement, FNAB or lymph node biopsies may be useful for the diagnosis.^3^

In the case above, FNAB and a cervical biopsy were done to exclude the possibility of neck metastases from local malignancies. Nasal endoscopy was extremely useful in detecting the primary lesion, and CT of the neck demonstrated the disease extension.

The differential diagnosis is made with lymphoreticular malignancies such as lymphomas, Hodgkin's disease, malignant histiocytosis and monocytic leukemia, all of which have similar histopathological features. Atypias in cytology and the aggressive clinical course establish the diagnosis in most cases. Other histiocytoses, such as rhinoscleromas, Wegener's granulomatosis, may also be included in the differential diagnosis.6 Serology for HIV, toxoplasmosis and syphilis was done since these conditions are not rare in our context. Extranodal manifestations in the head and neck are significantly more common in SHML patients with immunological abnormalities.4,5 SHML has been described in HIV-infected patients. An altered immune system and liver, kidney or lower airway involvement are considered as factors that worsen the prognosis.^3^

Immunohistochemical exams were not done in our patient; the public health system (Sistema Unico de Saude) did not authorize these tests. The diagnosis, therefore, was confirmed histologically.

The patient next underwent radiological and other image diagnostic tests (a chest X-ray, abdominal ultrasound and CT of the head) to investigate further extranodal involvement. The rhinopharyngeal lesion and diffuse skin lesions on the face were considered extranodal involvement of this disease. We did not correlate poor vision with the disease in this patient.

There is no ideal protocol for the treatment of SHML; it is an uncommon, self-limited disease that frequently requires no therapy. Treatment is only necessary when lymph node or extranodal tissue enlargement causes significant symptoms, such as airway obstruction or compression of vital organs. Pulsone at al. reviewed 80 cases published between 1969 and 2000; 50% of these cases required no treatment, of which 82% had full remission.^4^ The role of surgery is mostly for biopsies and to relieve obstruction.^3^ Local recurrence is frequent following surgical resection. The role of radiotherapy is not well understood; some reports have described full resolution with this treatment, while others have shown no response.^2^ Steroids often resolve fever and reduce lymph node size. Chemotherapy has yielded controversial results. A possible efficacy of methotrexate and 6-mercaptopurine requires further investigation. Other reports have suggested using alpha-interferon, although its side effects have limited its use.^4^

We treated our patient with corticosteroids, to which he responded well. The patient was sent to an oncology unit for chemotherapy, which was eventually not done. Surgery was not required, given the benign nature of the condition.

## FINAL COMMENTS

SHML is rare, requiring knowledge of its main clinical manifestations for a correct diagnosis. The differential diagnosis includes a variety of diseases. The diagnosis of SHML is made by histopathology; other exams may be useful. Therapy and when to begin treatment remains controversial. Follow-up is necessary to avoid relapses.
